# Except the unexpected: A TIPIC syndrome in a child with IPEX

**DOI:** 10.70962/jhi.20250246

**Published:** 2026-03-25

**Authors:** Vincent Fréret, Klervie Loiselet, Benjamin Terrier, Marie-Louise Frémond, Bénédicte Neven, Agathe Escudier

**Affiliations:** 1 Department of Paediatric Hematology-Immunology and Rheumatology, Necker-Enfants Malades Hospital, AP-HP, Paris, France; 2 Department of Pediatric Radiology, Necker-Enfants Malades Hospital, AP-HP, Paris, France; 3 National Referral Center for Rare Systemic Autoimmune Diseases, Université Paris Descartes, Hôpital Cochin, AP-HP, Paris, France; 4 Laboratory of Neurogenetics and Neuroinflammation, Imagine Institute, INSERM UMR1163, Paris, France; 5 Laboratory of Immunogenetics of Pediatric Autoimmune Diseases, Imagine Institute, INSERM UMR1163, Paris, France

## Abstract

A child with IPEX syndrome due to a *FOXP3* mutation presented with carotid perivasculitis consistent with TIPIC syndrome. This first pediatric report linking localized vascular inflammation to IPEX expands the recognized clinical spectrum of regulatory T cell deficiency.

## Teaser

This report by Fréret et al. describes a child with IPEX due to a FOXP3 mutation, presenting with carotid perivasculitis consistent with TIPIC syndrome on multimodal imaging, who achieved clinical and radiologic remission after corticosteroids. This represents the first pediatric case linking TIPIC and immune dysregulation.

## Introduction

Transient perivascular inflammation of the carotid artery (TIPIC) syndrome is a rare, benign, and usually self-limited condition characterized by acute unilateral cervical pain and focal perivascular inflammation of the carotid artery. Although reported in young to middle-aged adults without underlying systemic disease, occasional associations with autoimmune conditions have been described.

To our knowledge, TIPIC syndrome has never been reported in pediatric populations neither in immune dysregulation, polyendocrinopathy, enteropathy, X-linked (IPEX). We report a unique case documented by high-resolution imaging in a 10-year-old boy with IPEX, a rare monogenic disorder caused by *FOXP3* mutations impairing CD4^+^ regulatory T cell development and/or function. IPEX typically manifests with early-onset autoimmune enteropathy, endocrinopathies, and skin manifestations, though clinical presentations are highly variable. Cervical vascular involvement has not been reported previously in this inborn error of immunity. This case broadens the clinical spectrum of both rare conditions.

## Case presentation

A 10-year-old boy with IPEX syndrome related to a hemizygous pathogenic *FOXP3* mutation (c.1222G>A; p.V408M) presented with febrile torticollis. IPEX had been diagnosed at the age of 6 mo in the context of bullous pemphigoid. He also presented transient food allergies and allergic asthma and, at the age of 6 years, a nephrotic syndrome related to membranous glomerulonephritis in prolonged remission under sirolimus. He had no autoimmune enteropathy and endocrinopathy, and serial screens for autoantibodies remain negative. The mother, who is a heterozygous carrier for the p.V408M mutation, is healthy, as his father and young sister. Several cousins carrying the same hemizygous *FOXP3* mutation presented with skin or renal manifestations, diabetes, and atopic manifestations (asthma, food allergies).

1 mo before admission, the patient developed self-limited gastroenteritis shortly complicated by erythema nodosum, fever, polyarthralgia, and arthritis. He was treated with amoxicillin–clavulanate and a short course of corticosteroids. After corticosteroid withdrawal, fever recurred with nonspecific chest pain, low-grade systemic inflammation (C reactive protein [CRP] ∼15 mg/L), and subsequently acute left-sided neck pain radiating to the ear with reducible torticollis.

**Figure 1.**
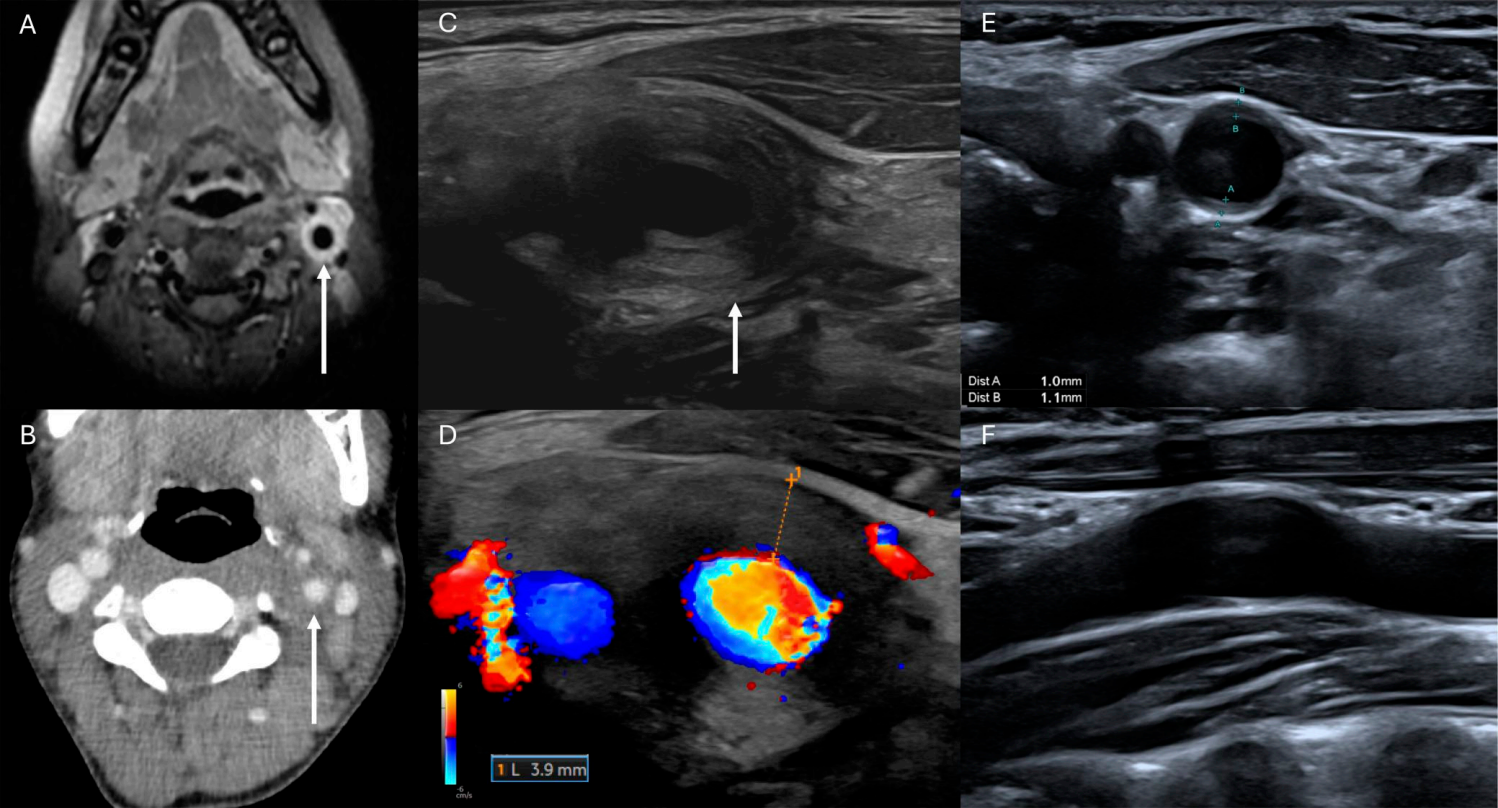
**Imaging of perivascular inflammation of the left internal carotid artery at diagnosis and at 4 wk of treatment. (A–F)** At diagnosis: circumferential parietal thickening (white arrows) of the left carotid artery wall on axial cervical views of MRI (A), CT scan (B), US (C), and Doppler (D). At midterm treatment: regression of wall thickening on axial (E) and sagittal (F) US views.

Computed tomography (CT) of the head and neck revealed circumferential parietal thickening of the left carotid artery wall (∼4 mm) with mass effect on the adjacent jugular vein, confirmed by ultrasound (US) and magnetic resonance imaging (MRI) (Fig. 1, A–D). Perivascular inflammation also extended to the left subclavian artery. No intracerebral, ocular, or cardiac vascular lesions were identified. Extensive infectious workup, including for syphilis, *Coxiella burnetii*, *Yersinia* spp., and fungi, was negative. Autoimmune tests were unremarkable (anti-nuclear antibodies, anti-centromere, anti-extractable nuclear antigen, anticardiolipin, anti-B2GP1 antibodies) except for mildly positive perinuclear anti-neutrophil cytoplasmic antibodies (80 U/ml) with no anti-PR3 or anti-myeloperoxidase (MPO) specificity. The interferon-γ release assay was negative.

Oral corticosteroids were restarted (2 mg/kg/day), leading to prompt resolution of fever, pain, and decreasing of CRP levels (from 16.9 to 3.7 mg/L) within 48 h. Treatment was tapered over 8 wk with complete clinical and radiologic remissions. Midterm US demonstrated a complete regression of carotid inflammation (Fig. 1, E and F) and resolution of inflammation in the left subclavian artery. After 18 mo of follow-up, the patient remained asymptomatic with no recurrence.

This unique and unusual presentation of perivasculitis of the left carotid artery fulfilled criteria for TIPIC syndrome, representing the first case reported in IPEX.

## Discussion

TIPIC syndrome, first reported in 1927, is a rare, self-limited vasculitis of carotid bifurcation clinically characterized by acute neck pain (also referred to as carotidynia) and radiologically by eccentric perivascular infiltration without significant endoluminal stenosis. Diagnosis criteria proposed by Lecler et al. in 2017 include: (1) acute unilateral cervical pain in regard to the carotid artery, (2) radiological evidence of perivascular inflammation, (3) exclusion of vascular and non-vascular causes of neck pain, and (4) improvement within 14 days either spontaneously or with anti-inflammatory treatment (1). Laboratory findings are typically unspecified (normal blood counts, and only occasional mildly elevated inflammatory markers) (1). Our patient fulfilled all these criteria with typical clinical and radiologic findings, without evidence of infectious or systemic large-vessel vasculitis.

TIPIC syndrome predominantly affects young to middle-aged adults, usually in the absence of underlying systemic disease. Only a few associations with autoimmune conditions have been described, including ankylosing spondylitis, Hashimoto thyroiditis, Graves’ disease, systemic lupus erythematosus, Sjögren’s syndrome, and rheumatoid arthritis (1). Importantly, no pediatric cases have been reported to date. Although TIPIC syndrome is self-limited with unknown pathophysiology, short courses of anti-inflammatory treatment such as nonsteroidal anti-inflammatory drugs, aspirin, or corticosteroids have been proposed, though no controlled studies exist.

To our knowledge, this is the first case of TIPIC syndrome reported in a patient with IPEX syndrome. IPEX is a primary immunodeficiency caused by mutations in the *FOXP3* gene located on the X chromosome. FOXP3 is a transcription factor involved in the development and function of CD4^+^CD25^+^ regulatory T cells, which play a critical role in maintaining immune tolerance and preventing autoimmunity. *FOXP3* hemizygous mutations lead in males to a severe, early-onset, immune dysregulation syndrome characterized by a wide spectrum of manifestations, such as enteropathy, endocrinopathies, and hematologic abnormalities, but also infections, cutaneous, and renal involvement (2). Of note, to our knowledge, autoimmune vasculitis has never been reported in IPEX patients.

A considerable heterogeneity exists in clinical presentation of IPEX patients. The mutation identified in our patient, i.e., c.1222G>A (p.V408M), located in exon 11, is associated with a milder phenotype and prolonged survival (3, 4). Functional studies suggest a less disruptive effect on FOXP3 protein structure, consistent with the milder course of the disease observed in these patients (3, 4, 5). Prolonged survival in this subgroup may provide sufficient time for the development of atypical autoimmune manifestations such as TIPIC syndrome.

Interestingly, in our patient the onset of carotidynia followed an acute episode of gastroenteritis, which was complicated by immune dysregulation manifestations such as erythema nodosa and arthralgia. This suggests that an infectious trigger may have acted as a facilitating factor on the background of the underlying FOXP3-related immune dysregulation, leading to localized vascular inflammation.

This unique case highlights the potential for atypical inflammatory vascular involvement in IPEX syndrome, suggesting shared immune-inflammatory pathways between both conditions and warranting further investigation into their underlying pathophysiology. Clinicians caring for IPEX patients should be aware of such unusual presentations, particularly in pediatric cases where TIPIC has not been previously described. Early recognition is crucial, as prognosis remains favorable with appropriate supportive and anti-inflammatory management. Multidisciplinary evaluation and systematic use of imaging are essential to ensure accurate diagnosis and optimal outcomes in these complex cases.

## Ethics

Written informed consent was obtained from the patient’s parents for publication of this case report and any accompanying images.
